# Improved Survival in Patients with Idiopathic Pulmonary Fibrosis Hospitalized for Acute Exacerbation

**DOI:** 10.3390/jcm14051693

**Published:** 2025-03-03

**Authors:** Federico Lionello, Giovanna Arcaro, Leonardo Bertagna De Marchi, Fausto Braccioni, Alessia Achille, Sara Lococo, Michele Ciresi, Gabriella Guarnieri, Andrea Vianello

**Affiliations:** Department of Cardiac, Thoracic, Vascular Sciences and Public Health, University of Padova, via Giustiniani 2, 35128 Padova, Italy; federico.lionello@aopd.veneto.it (F.L.); giovanna.arcaro@aopd.veneto.it (G.A.); leonardo.bertagnademarchi@aopd.veneto.it (L.B.D.M.); fausto.braccioni@aopd.veneto.it (F.B.); alessia.achille@aopd.veneto.it (A.A.); sara.lococo@aopd.veneto.it (S.L.); michele.ciresi@aopd.veneto.it (M.C.); gabriella.guarnieri@unipd.it (G.G.)

**Keywords:** idiopathic pulmonary fibrosis, acute exacerbation, nintedanib, pirfenidone, high-flow nasal oxygen, acute respiratory failure

## Abstract

**Background**: Patients suffering from idiopathic pulmonary fibrosis (IPF) may experience acute exacerbation (AE-IPF), which frequently results in acute respiratory failure (ARF) requiring hospitalization. **Objective**: This study aims to determine if survival has improved over the last decade in patients hospitalized for ARF consequent to AE-IPF, in view of the progress recently made in pharmacological and supportive treatment strategies. **Methods**: This was an observational retrospective single-center study. The data of 14 patients admitted to an Intermediate Respiratory Care Unit (IRCU) between 1 January 2004 and 31 December 2013 (group A) were compared with those of 26 patients admitted between 1 January 2014 and 31 December 2023 (group B). This study’s primary endpoint was survival following IRCU admission. **Results**: Survival time was significantly longer in the second group of patients compared to the first one [median survival time: 134 (31–257) vs. 25.5 (20–50) days; *p* < 0.001]. Group B patients also had a lower IRCU mortality rate (6/26 vs. 10/14; *p* = 0.003) and a significantly shorter stay in the IRCU [6 (1–60) vs. 14 (1–43) days; *p* = 0.039]. **Conclusions**: Innovative pharmacologic treatments and supportive therapeutic strategies are able to prolong survival and reduce the risk of in-hospital mortality in patients with AE-IPF hospitalized for ARF.

## 1. Introduction

A rare disease of unknown etiology, idiopathic pulmonary fibrosis (IPF) is characterized by progressive, irreversible scarring of the lung interstitium, leading to respiratory function decline and early mortality [1]. During the course of the disease, patients suffering from IPF may experience acute exacerbation (AE-IPF), which often results in acute respiratory failure (ARF), requiring hospitalization [2]. Crucially, approximately one-half of patients hospitalized with AE-IPF do not survive [3], and those admitted to Intensive Care Units (ICUs) have a nearly 90% mortality rate [4]. Worth mentioning, survivors have a life-expectancy significantly shorter compared to patients with IPF who do not experience an AE, ranging between 15.5 and 36 months [2,5,6].

Although progress over the past twenty years in the development of pharmacologic and supportive treatments for IPF has not always been linear, growing evidence has been underlining an improved survival rate in patients experiencing acute exacerbation (AE). Pirfenidone and nintedanib, respectively, approved for the treatment of IPF by the European Medicines Agency (EMA) in February 2011 and January 2015, have been associated with a decrease in in-hospital mortality [5,6,7]. Patients receiving pirfenidone were found, in fact, to have better three-month survival rate (55% vs. 34%) and a longer survival time (137.0 versus 16.0 days) compared to controls [7,8]. Moreover, subjects treated with nintedanib showed an in-hospital mortality rate significantly lower with respect to their counterparts (13.7% vs. 6.0%; OR: 0.43) [9].

The utilization of a corticosteroid and immunomodulator combination therapy was instead found to be associated with a worse prognosis during hospitalization [10] and discontinued after the ATS/ERS/JRS/ALAT released a consensus statement recommendation against its use in 2011 [5]. Several innovative supportive measures such as high-flow nasal oxygen (HFNO) therapy, extracorporeal membrane oxygenation (ECMO), and extracorporeal CO_2_ removal (ECCO_2_R), which were developed to improve oxygenation and reverse CO_2_ retention in the event of ARF, have been linked to promising results [4,11,12]. In view of these considerations, the current study aimed to determine if survival has improved over the last decade in patients admitted to an Intermediate Respiratory Care Unit (IRCU) for ARF due to AE-IPF.

## 2. Methods

This observational retrospective single-center study was conducted in a tertiary teaching hospital located in Northeast Italy. At admission, all the study participants were asked to sign informed consent forms agreeing to the use of their de-identified clinical data for research, analysis, and reporting purposes. Ethical approval was waived by the facility’s Institutional Review Committee in view of the fact that the study was retrospective in nature, and all the interventions prescribed were part of the hospital’s internal protocols (No.: 2772-30 December 2022). This study was carried out in accordance with the Declaration of Helsinki of 1975.

### 2.1. Patients

All the patients with IPF and AE who were admitted to the IRCU of the University of Padua Medical Center between 1 January 2004 and 31 December 2023 for ARF were considered eligible for our study. The criteria for the patients’ admission to our IRCU was failure of conventional O_2_-therapy (COT) to maintain SaO_2_ ≥ 92%. The patients’ diagnoses of IPF formulated in accord with the criteria proposed by the ATS/ERS/JRS/ALAT consensus statement [5] and later updates [13] were confirmed. The patients’ diagnoses of AE were confirmed in accordance with the recommendations of an international working group [14]. Patients who underwent lung transplant (LT) at a later date were excluded from this study.

The patients’ medical records, including their medical histories, demographic and clinical characteristics, and laboratory and pulmonary function data, were retrieved, reviewed, and analyzed. Pulmonary function data were obtained from pulmonary function testing (PFT) carried out in the six months prior to IRCU admission. The patients were divided into 2 groups depending on the decade during which they were admitted to the IRCU [i.e., 1 January 2004–31 December 2013 = group A or 1 January 2014–31 December 2023 = group B]. Their outcomes, calculated on the basis of their clinical status parameters at the time they were discharged from the IRCU and at the end of the follow-up period, as well as the number of days they remained in the IRCU, were analyzed.

### 2.2. Interventions

Between 2004 and 2013, patients with IPF attending our Medical Center were generally prescribed immunosuppressive therapy in the absence of contraindications. The therapy was no longer utilized after the ATS/ERS/JRS/ALAT consensus guideline recommendations [5] were released in 2011. Pirfenidone and nintedanib, which were, respectively, approved for the treatment of IPF by the Italian Medicines Agency (AIFA) in June 2013 and April 2016, were accordingly routinely prescribed to our patients with IPF but always following a frank discussion regarding the drugs’ potential side effects. High-dose corticosteroid therapy (prednisolone: 1 mg/kg/die) and broad-spectrum antibiotic regimens were administered to all the patients admitted to the IRCU over the entire study period.

COT, which was provided through standard non-rebreathing face masks, was the first-line supportive treatment that was utilized throughout the first decade of this study. In accordance with the center’s standard protocol, non-invasive ventilation (NIV) was provided to patients with exacerbated IPF showing signs of respiratory muscle fatigue or severe hypoxemia [15]. In the presence of contraindications to a prolonged use of NIV, they were shifted to invasive mechanical ventilation (IMV) by endotracheal intubation (ETI) unless they had already expressed the decision not to be intubated. A step-by-step protocol including the use of high-flow nasal oxygen (HFNO) as a second-line therapy in hypoxemic patients not responding to COT has been followed in our center since May 2013 [12]. Elective intubation and IMV following HFNO failure were not situations that were considered by the current treatment algorithm. Only patients on a waiting list for lung transplant (LT) were eligible for extracorporeal membrane oxygenation (ECMO). The strategies utilized as supportive treatments over the two decades analyzed by this study are outlined in Table 1.

### 2.3. Study Endpoints and Statistical Analysis

Survival following IRCU admission was considered the primary study endpoint. The mortality rate during the stay in the IRCU and the number of days in the IRCU were the secondary endpoints. The results were expressed, as appropriate, as mean values, ranges, and percentages. The continuous variables were compared, depending on the normality of the distributions, using Student’s t test or the Mann–Whitney U test. The categorical variables were compared, as appropriate, using the Chi-squared test or Fisher’s exact test. Survival from the time the patient was admitted to the IRCU was calculated using the Kaplan–Meier method; the log-rank test was used to compare the survival curves of the two groups. A bilateral *p* value < 0.05 was considered statistically significant for all the comparisons. All the statistical calculations were carried out using the Jamovy, Version 2.3, 2022 software.

## 3. Results

All forty-eight patients who were admitted to our IRCU with a diagnosis of AE-IPF during the study period (1 January 2004 to 31 December 2023) were considered eligible to participate in our retrospective study. The data of the eight patients who underwent LT at a later date were not included in the analysis. The age range of the patients included in the study was 44–83 years. Fourteen patients (group A) were admitted during the earlier decade and twenty-six (group B) during the later one. No group B patients had received immunosuppressive therapy before admission; meanwhile, six patients in group A had been administered azathioprine in combination with low-dose corticosteroids. Group B patients also showed better respiratory function (in particular, higher DLCO % values) (Table 2).

In group B, 11 patients were receiving pirfenidone at the recommended full dosage, i.e., three times daily at oral doses of 801 mg, while 15 were administered nintedanib at the twice-daily oral dose of 150 mg, prior to being hospitalized, and that dosage was continued throughout the time they were hospitalized. Survivors continued to receive the treatment after discharge from the hospital. When they were admitted to the IRCU, group B patients had significantly lower respiratory rates (RRs) and APACHE II score values and significantly higher PaO_2_/FiO_2_ values compared to their counterparts. Moreover, there were significantly fewer group B patients with plasma BNP levels above the reference range and CRP level > 100 μg/mL (Table 2). All patients received the appropriate supportive and pharmacologic interventions for ARF during their stay in the IRCU according to current internal protocols [12,15].

**Figure 1:**
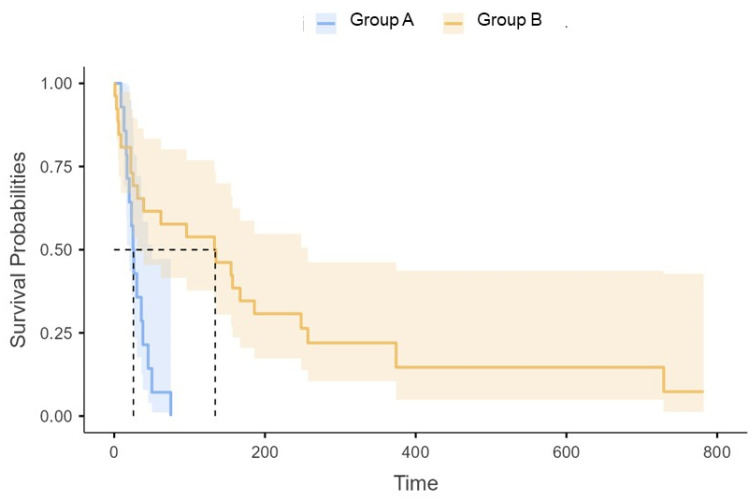
Kaplan–Maier estimates of survival function after Intermediate Respiratory Care Unit admission, stratified according to the group of origin. Dashed lines indicate median survival time.

The stratified log-rank test uncovered that group B patients survived for a significantly longer time compared to their counterparts [median survival time: 134 (31–257) vs. 25.5 (20–50) days; *p* < 0.001] (Figure 1); their hazard ratio for death was 0.27 (95% CI, 0.12 to 0.62, *p* = 0.002). Group 2 patients also had a lower IRCU mortality rate compared to their counterparts (6/26 vs. 10/14; *p* = 0.003). Finally, they spent significantly fewer days in the IRCU (Table 2).

## 4. Discussion

Although important advancements in the pharmacologic and supportive therapies used to treat patients with IPF and AE have been made over the past two decades, it is unclear if the outcomes of these patients have likewise improved. This study examined the question retrospectively by analyzing the survival of patients with exacerbated IPF admitted to our IRCU for ARF over the past two decades. Data analysis uncovered that the group of patients hospitalized more recently (after 2013) showed a significant improvement in survival times compared to those hospitalized during the previous decade. Indeed, their survival times had increased fivefold, and their IRCU mortality rate was also significantly reduced. Although it is difficult to pinpoint how the different interventions impacted the patients’ outcomes, several important considerations can be made.

(A) Impact of Antifibrotic Therapy

At the time of their IRCU admission, the patients belonging to group B (more recent) tended to have better lung diffusing capacity values compared to their counterparts. This finding led us to assume that their improved survival was linked, at least in part, to the fact that they had better preserved pulmonary function than their counterparts. Other studies have reported that AE is more severe when respiratory function declines [16]. Better respiratory function in group B patients could be explained by the impact of antifibrotic treatment on lung function. Indeed, it has been demonstrated that both pirfenidone and nintedanib effectively delay lung function deterioration in patients with IPF [17,18]. Group B patients also showed higher levels of PaO_2_/FiO_2_ at the time they were admitted to the IRCU. Hypothetically, a smaller decrease in PaO_2_/FiO_2_ values could reflect limited pulmonary parenchyma damage and less-severe AE-IPF [19]; for this reason, the better survival rates could be partially explained by reduced lung inflammation during the acute disease phase. In accordance with data from experimental models of lung fibrosis, nintedanib could inhibit the progression of lung inflammation [20,21]. In line with this hypothesis, a recent study by Urushiyama et al. showed that initiating nintedanib within 14 days of being admitted to a hospital for AE was significantly associated with a lower risk of in-hospital death in patients with fibrosing interstitial lung disease (ILD) [9]. Moreover, pirfenidone was found to suppress inflammatory cytokines such as TGF-b and basic fibroblast growth factor (b-FGF), which are related to lung inflammation during an acute phase and subsequent fibrosis progression [7]. Interestingly, there was a significantly lower number of patients with a CRP level consistent with severe inflammation (>100 μg/mL) [22] in group B patients.

(B) Avoidance of Immunosuppressive Medication

At the time of IRCU admission, no group B patient was receiving steroid and/or immunosuppressive medication compared to 6 out of the 14 patients in group A. Considering that a history of immunosuppression before AE-IPF may adversely influence patients’ survival [10,23], avoiding immunosuppressive therapy could have contributed to improved prognosis in patients hospitalized after 2013.

(C) Impact of Novel Supports for Oxygenation and CO_2_ Removal

A significant percentage of group B patients who were unable to achieve sufficient oxygenation using standard non-rebreathing face masks had a satisfactory response to HFNO, which was prescribed in accordance with an internal protocol [12]. As refractory hypoxemia due to marked ventilation/perfusion (V/Q) mismatch and impairment in lung diffusing capacity is a predictor of poor outcome in patients with exacerbated IPF [24], the patients’ satisfactory response to HFNO may have contributed to improving their survival. Indeed, the IRCU mortality rate was lower than 50% in the patients with AE-IPF who developed hypoxemia refractory to COT and showed a satisfactory response to HFNO [12]. A small single-center retrospective observational study likewise reported potential survival benefits in a cohort of 32 patients with ARF secondary to ILD treated with HFNO. That study found that the 30-day mortality was 23% as opposed to 63% in the patients receiving NIV [25]. We recently reported that a limited number of patients admitted to our IRCU over the past decade with severe CO_2_ retention refractory to NIV were successfully treated with ECCO_2_R as an alternative to IMV [26]. Since transitioning patients with AE-IPF to IMV is associated with a mortality rate of approximately 90% [27], ECCO_2_R may have also contributed to improving the patients’ survival.

This study has important limitations given its single-center, retrospective, and observational nature. Its population was also quite small, which is generally the case for clinical studies focusing on rare diseases and/or conditions. Its most important strength was that both IPF and AE-IPF were diagnosed following standard internationally accepted recommendations.

## 5. Conclusions

Despite its limitations, the preliminary results reported here corroborate the hypothesis that innovative pharmacologic and supportive treatments may prolong survival in patients with IPF hospitalized for AE. This strongly calls for collaborations between IPF specialists to develop novel clinical guidelines recommending the optimal management of this deadly complication.

## Figures and Tables

**Table 1:** Supportive treatments available to treat the patients admitted to the Respiratory Intermediate Care Unit during the two decades studied. ECMO was considered only for those patients on a waiting list for lung transplant. (COT = conventional oxygen therapy released through non-rebreathing face masks; ECCO_2_R = extracorporeal CO_2_ removal; ECMO = extracorporeal membrane oxygenation; ETI = endotracheal intubation; HFNO = high-flow nasal oxygen; IMV = invasive mechanical ventilation; NIV = non-invasive ventilation; and RMF = respiratory muscle fatigue).

	Group A (Admission: 2004-13)	Group B (Admission: 2014-23)
**First-line treatment**	COT	COT
**Refractory hypoxemia**	NIV	HFNO
**Hypercapnia, signs of RMF**	NIV	NIV
**NIV failure**	IMV via ETI	ECCO_2_R
**HFNO failure**	-	ECMO

**Table 2:** Patients’ baseline demographic, clinical, and pulmonary–cardiac function characteristics and laboratory data at the time of their admission to our Intermediate Respiratory Care Unit and their outcomes. (ACCI = Age-Adjusted Charlson Comorbidity Index; APACHE = Acute Physiology and Chronic Health Evaluation; BMI = body mass index; BNP = B-type natriuretic peptide; CRP = C-reactive protein; DLCO = diffusing capacity for carbon monoxide; FVC = forced vital capacity; FEV_1_ = forced expiratory volume in the first second; GCS = Glasgow Coma Scale; IRCU = Intermediate Respiratory Care Unit; OCSs = oral corticosteroids; PaO_2_/FiO_2_ = ratio of arterial oxygen tension to inspired oxygen fraction; PCT = procalcitonin; and PH = pulmonary hypertension).

	**Group A** **(Admission: 2004-13)** **(n = 14)**	**Group B** **(Admission: 2014-23)** **(n = 26)**	*p* Value
***Baseline demographic and clinical data***			
Age (years), mean (range)	71.6 (10.8)	68.3 (10)	0.33
Gender (male/female)	7/7	21/5	0.55
BMI (kg/m^2^), mean (range)	24.3 (3.2)	24.7 (3.1)	0.75
Smokers, n (%)	10 (71.4)	21 (80.8)	0.69
Length of time from diagnosis to IRCU admission (yrs), mean (range)	2.0 (0.2–6.0)	2.2 (0.0–9.0)	0.95
Pts with comorbidities, n (%)	11 (78.6)	24 (92.3)	0.32
ACCI score, median (range)	5 (1–6)	4 (2–12)	0.38
Pts with cancer disease, n (%)	2 (14.3)	2 (7.7)	0.50
Pts administered immunosuppressive therapy, n (%)	6 (42.8)	0 (0)	<0.001
Pts administered OCS therapy, n (%)	6 (42.8)	0 (0)	<0.001
Pts administered antifibrotic therapy, n (%) - Pirfenidone - Nintedanib	0 (0) 0 (0)	11 (42.3) 15 (57.7)	<0.001
Pts receiving home oxygen therapy, n (%)	7 (50)	14 (53.8)	0.71
FVC, L mean (range)	1.41 (0.73–2.94)	1.82 (0.95–2.75)	0.37
FVC, % mean (range)	51 (21–107)	55 (36–101)	0.99
FEV_1_, L mean (range)	1.25 (0.72–2.70)	1.70 (0.91–2.66)	0.25
FEV_1_, % mean (range)	63 (24–113)	68 (41–120)	0.92
DLCO, mL/min/mmHg, mean (range)	3.42 (1.04–13.00)	5.95 (2.33–8.38)	0.10
DLCO, % mean (range)	15 (5–51)	28 (11–49)	0.050
Pts with PH, n (%)	2 (14.3)	10 (38.5)	0.11
GAP Index, mean (range)	4 (2–7)	5 (1–7)	0.38
***Clinical, laboratory, and blood gas data at the time of IRCU admission***			
Respiratory rate (breaths/min), median (range)	32 (22–47)	25 (25–47)	0.003
Heart rate (beats/min), mean (range)	104 (86–170)	99 (58–130)	0.097
GCS media (range)	15 (12–15)	15 (12–15)	0.21
Pts with fever (temperature > 38 °C), n (%)	6 (42.9)	5 (19.2)	0.11
Pts with leukocytosis (WBC > 12,000 × 10^6^/L), n (%)	10 (71.4)	12 (46.2)	0.12
PaO_2_ * (mmHg), mean (range)	64.8 (34.0–97.5)	80.0 (39.0–317)	0.051
PaCO_2_ (mmHg), mean (range)	46.9 (25.3–90.0)	39.4 (27.5–82.3)	0.13
Arterial pH, mean (range)	7.40 (7.25–7.49)	7.42 (7.26–7.51)	0.56
PaO_2_/FiO_2_ (mmHg), median (range)	100 (35–268)	173 (46–421)	0.002
Pts with abnormal BNP level, n (%)	8 (57.1)	8 (30.8)	0.011
CRP (μg/mL), mean (range)	86.3 (2.9–181.0)	38.0 (2.1–395.0)	0.55
Pts with CRP level > 100 μg/mL, n (%)	4 (28.6)	1 (3.8)	0.042
Pts with PCT level > 0.5 μg/L, n (%)	1 (7.1)	4 (15.4)	0.92
APACHE score, median (range)	21 (14–26)	13 (5–33)	0.032
***Clinical outcomes***			
Patients who died during IRCU stay, n (%)	10 (71.4%)	6 (23.1)	0.003
Length of IRCU stay, days	14 (1–43)	6 (1–60)	0.039

* during supplemental O_2_-therapy

## Data Availability

The data presented in this study are available upon request from the corresponding author. The data are not publicly available due to privacy restrictions.
